# Not one, but many: developing a multi-indication pricing model for medicines in Belgium

**DOI:** 10.3389/fphar.2023.1199253

**Published:** 2023-09-28

**Authors:** Ingrid Maes, Eline Kok, Pieter-Jan De Torck, Jorge Mestre-Ferrandiz, Steven Simoens

**Affiliations:** ^1^ Inovigate, Wilrijk, Belgium; ^2^ Independent Economics Consultant, Madrid, Spain; ^3^ KU Leuven Department of Pharmaceutical and Pharmacological Sciences, Leuven, Belgium

**Keywords:** multi-indication pricing, pricing models, typology, (dis)advantages, implementation, pathway, Belgium

## Abstract

**Back ground:** Current pricing and reimbursement models that focus on one indication at a time are not suited to address the market access of multi-indication medicines. Therefore, the aim of this study is to co-create with Belgian stakeholders a multi-indication pricing model and procedural pathway, to identify conditions for implementation, and to illustrate the multi-indication pricing model with a case study.

**Methods:** Different multi-indication pricing models were identified from the literature, case studies and pilots in other countries. Semi-structured interviews were conducted with 21 representatives from the National Institute for Health and Disability Insurance, insurance funds, clinicians, patients, the policy cell of the Minister of Health, pharmaceutical industry and academia. These provided insight in the opinions of stakeholders about possible multi-indication pricing models and their feasibility in the Belgian context. Agreement on the preferred multi-indication pricing model and procedural pathway was reached in a multi-stakeholder round table.

**Results:** The international review generated four main multi-indication pricing models that vary in terms of whether a uniform price or differential prices are applied, whether prices are adjusted for the volume and/or value of the medicine in each indication, and whether a proactive or retroactive dynamic pricing approach is used. However, Belgian stakeholders preferred a fifth model, which sets a single price as the volume- and value-weighted average price across all indications at launch. Over time, the price is adapted based on volume and value of the medicine in real-life practice for each indication. To implement this model, a legal framework, horizon scanning and early dialogue, data infrastructure, an evidence plan for the medicine, technical expertise and governance model need to be developed.

**Conclusion:** Although the multi-indication pricing model preferred by Belgian stakeholders raises the administrative burden, it allows for the price of a medicine to vary during the lifecycle based on its initial and real-life performance in multiple indications.

## 1 Introduction

Medicines can have many uses beyond their first authorisation for a specific indication. Broadly speaking, there are three types of additional indications: across distinct therapeutic areas (e.g., oncology and ophthalmology); across different disease areas, but within the same therapeutic area (e.g., different types of cancers); and across different lines of therapy, but within the same therapeutic and disease area (e.g., first line vs. second line in a specific cancer) (Michaeli et al., 2022a). This is not a new phenomenon, and indeed has been discussed and analysed at different levels for some years now. Moreover, this trend is perhaps more perceptible in the oncology field, where the existence of subpopulations and several closely connected indications implies that it is particularly prone to multi-indication medicines. As an illustration, there was a 50% increase in the share of oncology medicines with more than one indication authorized from 2014 to 2021 (Lawlor et al., 2021). This increase has been related to the discovery of immune checkpoint pathways and the associated developments in immuno-oncology medicines (Pan et al., 2020).

The issue at stake is how to assess, price and reimburse the new indications of a medicine-indication combination with an existing pricing and reimbursement status, or a new medicine-indication combination with future additional indications in the pipeline. Importantly for our purposes, the value delivered in each of the indications, irrespective of how value is measured (Landon et al., 2021), can be different. Reimbursing additional indications also raises financial challenges to already-financially constrained healthcare systems. A question for countries is then if, and how, they would like to treat the new indications within their health technology assessment, pricing and reimbursement processes. There are various options, and indeed countries use different processes and mechanisms for the follow-on indications. However, most countries adopt a single-price-per-molecule approach, de-linking the overall price from the value that individual therapeutic indications provide (Michaeli et al., 2022a). As discussed in detail later, this single price, and its evolution over time, is determined according to different criteria. The other option is to have different prices per indication. Again, how to set different prices depends on different criteria, including differentiation between the “official” or list price of the medicine (which tends to be public) and the “net” price once discounts are included (which is usually not publicly available).

As a result of the importance of multi-indication medicines, a variety of studies have theoretically analysed and reviewed multi-indication medicines regarding economic frameworks, healthcare evaluations, feasibility of implementation across countries, or single cases (Michaeli et al., 2022b). There is, however, a lack of evidence about the value and pricing of multi-indication medicines, except for the research conducted by Michaeli et al. (Michaeli et al., 2022a; Michaeli et al., 2022b). These authors explored the assessments, value and pricing of 25 multi-indication cancer medicines across 100 indications (25“initial” and “75supplementary” indications) approved by the Food and Drug Administration between 2009 and 2019 across seven countries (England, Scotland, France, Germany, Canada, Australia, and the United States). Results demonstrate that manufacturers seem to prioritise and first launch orphan indications offering significant health gains and targeting serious diseases with high unmet medical need, while regulatory agencies use special review pathways to prioritise resources towards indications which they believe offer significant value to patients. These indications are then extended to ones that deliver lower health gains to more eligible patients.

There is also evidence reflecting the disparity of views and understanding of multi-indication pricing (MIP) and its implications (Cole et al., 2020). This is contrary to the vision that a prerequisite of meaningful progress towards any kind of pricing reform is a shared understanding of what MIP is. It seems that the pharmaceutical industry has the best understanding of MIP, followed by payers and regulators, while patient groups have the least. Lack of understanding is clearly a challenge for an informed debate on the use of MIP (Cole et al., 2020).

In the past, Belgium has implemented measures to address the issue of MIP. A first measure applied linear price cuts when new indications of a medicine were approved (as is the case in many other countries). This was informed by the rationale that increased use of the medicine needs to translate into a reduced price. However, and as argued above, such a measure suffers from the limitation that the medicine’s price is not linked to its value per indication. The Pact of the Future, which the Minister of Health agreed with pharmaceutical industry in 2015, stipulated that a new MIP model needed to be developed which promotes innovation and which relates pricing to a medicine’s clinical value and number of patients treated (De Block).

In September 2018, the National Institute for Health and Disability Insurance published guidelines that included a decision tree and a formula to determine the multi-indication price for a medicine (National Institute for Health, 2018). Although the tree considers the added therapeutic value of the medicine in the new indication, a major feature of the decision making process is to set a multi-indication price that restricts the incremental healthcare budget impact associated with the new indication. The multi-indication list price is calculated as the weighted average of the prices (accepted by the Drug Reimbursement Commission) for the various indications. Alternatively, a managed entry agreement can be negotiated for a new indication for which there is a therapeutic or social need. An advantage of this MIP model is that it allows to set a single list price, while having different (net) prices per indication. However, when a new indication is approved, this model requires complex and time-consuming negotiations to take place between the Drug Reimbursement Commission and the company. Also, the launch sequence of indications can have a large impact on the price. Furthermore, from an industry perspective, the current model stimulates companies to develop small-volume, high-value indications. This historical experience underlines the need to further explore MIP models.

The key objective of this manuscript is to provide an analysis of potential MIP models that could be applied in Belgium for multi-indication medicines. For this purpose, the manuscript distinguishes between different MIP models and reviews their challenges and (dis)advantages, elicits the MIP model preferred by Belgian stakeholders and illustrates this with a case study, identifies prerequisite conditions and elaborates a procedural pathway for implementing the preferred MIP model in Belgium. This analysis of MIP models is general and hopes to improve the level of understanding that stakeholders have of MIP inside and outside Belgium.

## 2 Materials and methods

A multi-method and multi-step study design was adopted with a view to co-create with Belgian stakeholders a MIP model and procedural pathway for Belgium, consisting of a literature review, semi-structured interviews and a round table. In accordance with the Belgian law of 7/5/2004 concerning experiments on human people, approval by an ethics committee was not required.

### 2.1 Literature review

A review of the literature was carried out to identify the various MIP models, their pros, cons and implementation challenges, as well as to investigate case studies and pilots in other countries. Using combinations of the key terms “multi indication”, “indication based”, “indication specific”, “pricing” and “medicines”, several searches were conducted in Web of Science (including Medline) for the peer-reviewed literature for the 2020–2022 period. A recent review on the topic was published in 2022 (Preckler and Espin, 2022), but needed an update as it included papers published up to and including 2019. Also, the grey literature was searched using the same key terms in Google, reviewing the first 30 hits. Each included reference was summarised, using the general following themes: pros, cons, implementation challenges, options discussed, and any other information relevant for this manuscript.

### 2.2 Belgian stakeholder views on MIP models

Informed by the literature review, one-on-one semi-structured interviews with Belgian stakeholders in 2021 collected their opinions about different MIP models and discussed the feasibility of these models within the Belgian context. Stakeholders included 21 representatives from the National Institute for Health and Disability Insurance, insurance funds, clinicians, patients, the policy cell of the Minister of Health, pharmaceutical industry and academia. The interview guide elicited: a) the perspective of stakeholders on the problem; b) suggestions and criteria for a MIP solution; c) perception of and preferences for MIP models; and d) initiatives and actions to be undertaken for MIP implementation. Third, an information session about the MIP problem and possible solutions was held in January 2022. Finally, a multi-stakeholder round table reached consensus on the preferred MIP model and procedural pathway for Belgium in June 2022.

### 2.3 MIP case study

To illustrate how the MIP model preferred by Belgian stakeholders can be operationalised, a hypothetical case study was developed. This case study pertains to a medicine which has two high-value, low-volume indications at time point T0 and an additional lower-value, higher-volume indication at T2. The choice of this sequence of indications is informed by recent evidence indicating that multi-indication cancer medicines first launch high-value, low-volume indications (Michaeli et al., 2022a; Michaeli et al., 2022b). The evolution in the number of patients and value-based price over time per indication is summarised in Table 1. To make the case study more realistic, it is assumed that the number of patients for indications 1 and 3 increases by 5% per year, while it remains constant over time for indication 2. Also, the case study explores the impact of changes in the value-based price over time for indication 3: two scenarios are developed in which this price increases and decreases, respectively, from T4 onwards based on evidence about the value of the product in real-life clinical practice.

**TABLE 1:** Input data over time for case study on MIP model preferred by Belgian stakeholders.

Parameter	T0	T1	T2	T3	T4	T5
Number of patients in indication 1	5,500	5,775	6,064	6,367	6,685	7,020
Value-based price for indication 1	2,000	2,000	2,000	2,000	2,000	2,000
Number of patients in indication 2	1,000	1,000	1,000	1,000	1,000	1,000
Value-based price for indication 2	2,500	2,500	2,500	2,500	2,500	2,500
Number of patients in indication 3			25,000	26,250	27,563	28,941
Increasing value-based price for indication 3			1,500	1,500	1,750	1,750
Decreasing value-based price for indication 3			1,500	1,500	1,250	1,250

Note: T, time point.

## 3 Results

### 3.1 Key findings from literature review

The literature review identified 77 hits, excluding duplications. After review of abstract, and full paper when in doubt, 16 references were included (seven references from Web of Science and nine references from the grey literature) (Pearson et al., 2016; Campbell, 2017; Baker et al., 2018; Cookson, 2019; Lawlor et al., 2021; Pereira et al., 2021; Creating; Adida, 2022; Michaeli et al., 2022a; Michaeli et al., 2022b; Gill et al., 2022; Ha et al., 2022; Pani et al., 2022; Preckler and Espin, 2022; Vokinger and Kesselheim, 2022; Zhang et al., 2022). This section summarises the main messages from the literature review, focusing on MIP typologies, (dis)advantages and implementation challenges. Some examples from specific countries are presented too.

#### 3.1.1 Typologies

Conceptually, and assuming that the list price is the same as the net price, there are two extreme models for setting prices for multi-indication medicines: a single price for all indications (“pure” uniform pricing) or one price per indication (“pure” multi-indication pricing). Between these two extremes there are various possibilities, including having a “uniform” list price for all indications, but different ‘net’ prices, as well as having different brands for the same molecule. In practice, however, MIP-type arrangements have been operationalised in a number of ways, but broadly speaking, there are three options related to how to set the price(s), the first one being a “pure” MIP and the last two being “indirect” or “blended” MIP models:

1. Different indications can be authorised and marketed under different brand names and prices (“pure” MIP).

This model could work for a medicine with indications across distinct therapeutic areas [for example, a product for multiple indications in oncology and ophthalmology with different dosage forms, or a product for onco-haematology and multiple sclerosis (Mestre-Ferrandiz et al., 2015)]. There is still a risk of arbitrage if there are large price differences between different indications. Nevertheless, this option is less relevant for this work, and is relatively rare, so it is discarded as a future option for Belgium.

2. There is a “blended” single price for the various indications, estimated as an average, with two variants on how to estimate the average: a) weighted only by expected volumes of use in each indication (i.e., prices not based on value) or b) weighted reflecting volumes of use and value of each indication (i.e., prices partially reflecting value).

A weighted average price can be calculated using estimates of the population size for the different indications. A retrospective review can help make adjustments based upon actual use across indications. This model is simpler but requires robust data capabilities, which is probably why many countries have opted for this blended/weighted price approach, whereby the price of a therapy is re-negotiated upon introduction of a new therapeutic use. Examples include Germany which negotiates the price for each new indication and those negotiations are then reflected in a final common price, and France where individual negotiations for each indication are scored according to the therapeutic benefit and the public price reflects the weighting of the indication by expected volume. However, no consensus exists on the best method of determining the weighted price (Gill et al., 2022). Also, under this blended price model, the prescriber and budget holder face an average price and not the price that is relevant for the indication for which they plan to use the product (Towse et al., 2018).

3. A single list price, with differential adjustments of net prices, aligning them to a value-based payment model per individual indication (blended MIP model partially reflecting value).

This model uses a single list price, but entails different net prices per indication in the context of financial- or outcome-based managed entry agreements. As this model is based on a list price, it does not impact prices in other countries that apply external reference pricing. Use across indications needs to be tracked and payment/reimbursement mechanisms implemented. Outcomes-based payments specifically can be difficult to negotiate and implement, as they require agreement on which outcomes to track and whether patients achieve them. Also, the list price could reflect the indication with the greatest value, with differential discounts for indications that provide less value. Netherlands, for example, opted to establish a price for the first indication, which is used to anchor the price of all further indications. If further indications are not cost-effective (at the prevailing price), either a lower price is negotiated or the medicine may not be reimbursed (Pereira et al., 2021). Italy uses a large registry with individual patient data (thus requiring extensive data systems), and the UK reviews each indication separately and recommends it if it is shown to be clinically and cost-effective (Pereira et al., 2021).

In addition, and beyond these three models to set (list or net) prices for multi-indication medicines, countries use two mechanisms targeting the demand side to differentiate between indications. On the one hand, clinical restrictions and guidance directed towards clinicians can limit prescription to subpopulations of subsequently launched indications. On the other hand, insurers/third-party payers may place one indication in a preferred tier for patients, and place the same medicine in a less preferred tier (or forego coverage altogether) for a different indication (in countries with different co-payment tiers).

#### 3.1.2 (Dis)advantages

Tables 2, 3 summarise the advantages and disadvantages, respectively, from MIP models identified from the literature review. In summary, MIP can increase static efficiency (i.e., optimal use of medicines) in the short term given that all patients get access to cost-effective treatments and benefit from them. In the long term, MIP increases dynamic efficiency (i.e., optimal investment in R&D) given that every indication developed is appropriately priced according to its value and manufacturers can recover R&D investments (Preckler and Espin, 2022). However, the operation of MIP models requires an appropriate data collection infrastructure, increases the administrative burden, may lead to arbitrage or actually have a negative impact on patient access.

**TABLE 2:** Advantages of MIP models Pearson et al., (2016); Campbell, (2017); Baker et al., (2018); Cookson, (2019); Lawlor et al., (2021); Pereira et al., (2021); Adida, (2022); Michaeli et al., (2022a); Michaeli et al., (2022b); Gill et al., (2022); Ha et al., (2022); Pani et al., (2022); Preckler and Espin, (2022).

Advantage	Description
Better alignment of medicines’ value and price	- Indications reimbursed at a level considered as cost-effective, representing good value for money for healthcare systems
	- Payers ensure they only pay for the benefits generated by medicines
Improved and faster patients’ access to treatment	- New indications encouraged irrespectively of existing prices
	- Can stimulate R&D in new high-value indications despite a moderate price obtained in a first lower-value indication
Improved transparency in pricing system	- Helps “rationalise drug pricing”, and makes negotiation more flexible which could lead to reduced prices for lower-value indications
Optimisation of R&D incentives	- Aids decision making regarding pipeline prioritisation
Increased competition	- Can generate price competition across the different indications
Increased collaborations and novel mechanisms	- Commitment by all parties to consider overall health system costs and financial sustainability
	- Highlights competitive advantage in innovative thinking about value-based pricing mechanisms

Source: authors’ summary of literature review. Note: MIP, multi-indication pricing.

**TABLE 3:** Disadvantages of MIP models Pearson et al., (2016); Lawlor et al., (2021); Pereira et al., (2021); Adida, (2022); Michaeli et al., (2022a); Michaeli et al., (2022b); Gill et al., (2022); Ha et al., (2022); Pani et al., (2022); Preckler and Espin, (2022).

Disadvantage	Description
Monitor, tracking and payment requirements	- Requires accurate monitoring, prescription tracking, and paying for products per indication
	- Appropriate data collection and infrastructures are needed
	- Differential discounts require the ex-post tracking of use per indication
Risk of greater administrative burden and associated costs	- For purchasing and payment process
	- Monitoring and registering of the specific use per indication
Risk of arbitrage	- Buying the medicine at the lowest price when it is used for the higher-value (and thus higher price) populations
Increase in prices and budget impact	- In the short term, all the consumer surplus is captured by manufacturers (but will depend upon the value and price of the first marketed indication)
Communication issues	- Could be difficult to explain to patients and stakeholders and may raise concerns if not tied to lower out of pocket costs for patients
Unequal access across indications	- Could limit a given medicine’s usage among certain subpopulations and/or increase patient discrimination
Access delays	- Negotiations for a specific indication can be delayed if further indications are expected soon (to negotiate collectively)
Challenges around value-based pricing systems	- Intrinsic challenges of value-based pricing systems apply, such as value metrics and price setting mechanisms

Source: authors’ summary of literature review. Note: MIP, multi-indication pricing.

#### 3.1.3 Implementation challenges

There is a general consensus in the literature on the challenges of implementing MIP models (see Table 4). Data and regulatory hurdles are described as the principal challenges in almost every country, but these are not the only ones mentioned. Partly to address these challenges, it is necessary to assess whether *ad hoc* registries need to be created (with the associated delays and costs) or whether it is possible to adapt current medical data systems to include indication-specific information.

**TABLE 4:** Implementation challenges of MIP models [Michaeli et al., (2022a); Lawlor et al., (2021); Michaeli et al., (2022b); Preckler and Espin, (2022); Adida, (2022); Baker et al., (2018); Campbell, (2017); Cookson, (2019); Gill et al., (2022); Ha et al., (2022); Pani et al., (2022); Pearson et al., (2016); Pereira et al., (2021); Creating].

Challenge	Description
Insufficient data systems and analytic capabilities	Data collection and data infrastructure need to improve, as currently it may be difficult to identify the use to which a product has been put, and to update periodically volume and pricing data
Legal or regulatory hurdles	Specific country legislations that hinder its application, including a public payer being only allowed to set one price for a medicine, or having to link the discounts given in the private sector and the price that must be offered to the public sector (e.g., previously, the US Medicaid “best price” rule implied giving Medicaid the lowest indication price offered to any purchaser (with exceptions), across all indications in exchange for Medicaid coverage)
Lack of stakeholder buy-in and political will	There is currently scarce number of MIP contracts seen in practice and a lack of understanding about this system
Complex medicine purchasing and delivery systems, generating contractual or financial flow issues	The complex distribution system has a number and variety of intermediaries existing between the manufacturer and the dispenser. This is because in most systems the manufacturer does not sell the medicine directly to the care providing institution or clinician prescribing the drug
Integration of MIP in co-payment systems	It can be difficult to link indication-specific pricing to differential patient cost sharing in countries with co-payment tier structures

Source: authors’ summary of literature review. Note: MIP, multi-indication pricing.

### 3.2 Belgian stakeholder views on MIP models

**FIGURE 1:**
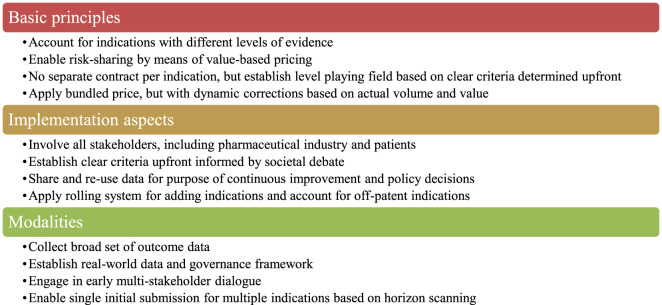
Design criteria for a preferred MIP model identified by Belgian stakeholders. Note: MIP, multi-indication pricing.

Belgian stakeholders identified a number of design criteria that a preferred MIP model should meet. These criteria were structured into four basic principles, four implementation aspects and four modalities (see Figure 1). Overall, it was emphasised that the implementation of a preferred MIP model requires real-world data access and a rolling system to adjust price per indication in a bundle.

**FIGURE 2:**
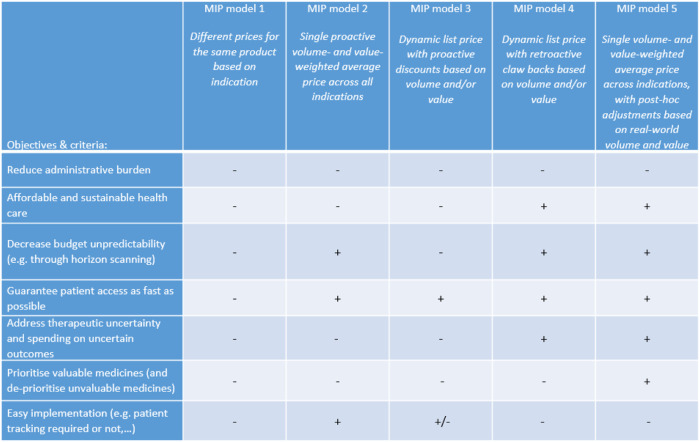
Belgian stakeholder preferences for MIP models. Note: MIP, multi-indication pricing.

For discussion with Belgian stakeholders and following the review of the literature, four MIP models were put forward that differ in terms of the application of uniform or differential pricing, price adjustment based on volume and/or value, proactive or retroactive dynamic pricing. Table 5 presents a description of each model and its (dis)advantages as perceived by our sample of Belgian stakeholders. The preferences of stakeholders for these MIP models based on the objectives and design criteria of Figure 1 are visualised in Figure 2. These results indicated that stakeholders prefer a new MIP model (i.e., model 5) combining features of model 2 (a single proactive volume- and value-weighted average price across all indications) and model 4 (a dynamic list price with retroactive claw backs based on volume and/or value).

**TABLE 5:** Description of MIP models and their (dis)advantages as perceived by Belgian stakeholders.

Model	MIP model 1	MIP model 2	MIP model 3	MIP model 4
	*Different prices for the same product based on indication*	*Single proactive volume- and value-weighted average price across all indications*	*Dynamic list price with proactive discounts based on volume and/or value*	*Dynamic list price with retroactive claw backs based on volume and/or value*
Description	The product is marketed under different pack presentations for different indications, resulting in differentiated prices	Weighted average price is proactively calculated based on volume and value of each indication	Starts from a single price based on indication with the lowest volume and/or value	Starts from a single price based on indication with the lowest volume and/or value
		A single price is set for all indications	Differential discounts are applied proactively based on their relative volume and/or value with respect to the lowest volume/value indication	Differential claw backs are applied retroactively based on their relative volume and/or value with respect to the lowest volume/value indication
		Can be implemented in context of so-called “multi-year, multi-indication” agreement Lawlor et al., (2021)	Can be implemented in context of financial managed entry agreement	Claw backs are claimed based on real-world volume and/or value of the product
Examples	Sildenafil: marketed as Viagra^®^ for erectile dysfunction and as Revatio^®^ for pulmonary arterial hypertension	Vertex’s cystic fibrosis treatments in Denmark, Ireland and Sweden	Volume-based discounts for tisagenlecleucel	-
			Value-based discounts applied by individual insurers for dupilumab in Australia, United States and Germany	
(Dis)advantages perceived by Belgian stakeholders	• Challenging to track patients and negotiate a different price per indication in absence of required data infrastructure and capability	• Potential to under/over-estimate the price, but can be remediated at end of managed entry agreement• The launch sequence of indications does not impact price • Less administrative burden, as one dossier is required for bundle of indications	• Proactive design guarantees revenues for companies and budget impact for payer • Complex implementation as patients need to be tracked	• Revenues for companies and budget impact for payer are uncertain • Complex implementation as patients need to be tracked • Requires infrastructure and capabilities for real-world data access
	• The launch sequence of indications may have large impact on price			
	• High administrative burden, as separate dossier needs to be prepared and evaluated for each indication			
	• Perceived to be an artificial solution, not supported by payer or industry			

Note: MIP, multi-indication pricing.

### 3.3 MIP model preferred by Belgian stakeholders

In MIP model 5, an initial price is calculated as the volume- and value-weighted average price across all indications that are available at launch. This single price then evolves over time on the basis of real-world volume and value of the medicine for each indication. In other words, the price increases when the medicine provides more value or when it reaches a lower sales volume than anticipated and *vice versa*. If a new indication is added, the single price is adjusted based on available volume and value data for that indication. Belgian stakeholders argued that the initial price should be set lower than the volume- and value-weighted average price across indications as, in their opinion, value at launch can sometimes be difficult to estimate. The implementation of this MIP model requires that a legal framework, horizon scanning, data infrastructure, technical expertise and governance model are in place with a view to regularly adjust the price based on the real-life performance of the medicine. Finally, as it can be expected that real-world volume and value of the medicine in each indication becomes more stable after a number of years, the single price can be fixed or adjusted over a longer interval, thereby reducing administrative efforts.

Belgian stakeholders argued that MIP model 5 is particularly suitable when evidence about the volume and value of the medicine for indication(s) at launch is unknown (and it is hence difficult to set the initial price) or uncertain (and additional real-world evidence needs to be collected). When sufficient evidence about the volume and value of the medicine is available, stakeholders thought that model 2 is fit for purpose.

Although MIP model 5 was preferred by Belgian stakeholders, they acknowledged some shortcomings (see also Figure 2). In particular, this MIP model does not necessarily lead to better budget predictability, but to more control of the budget. Additionally, it was thought that MIP model 5 is less attractive for pharmaceutical industry and might lead to fewer market introductions, except for medicines with high uncertainty.

### 3.4 MIP case study

**FIGURE 3:**
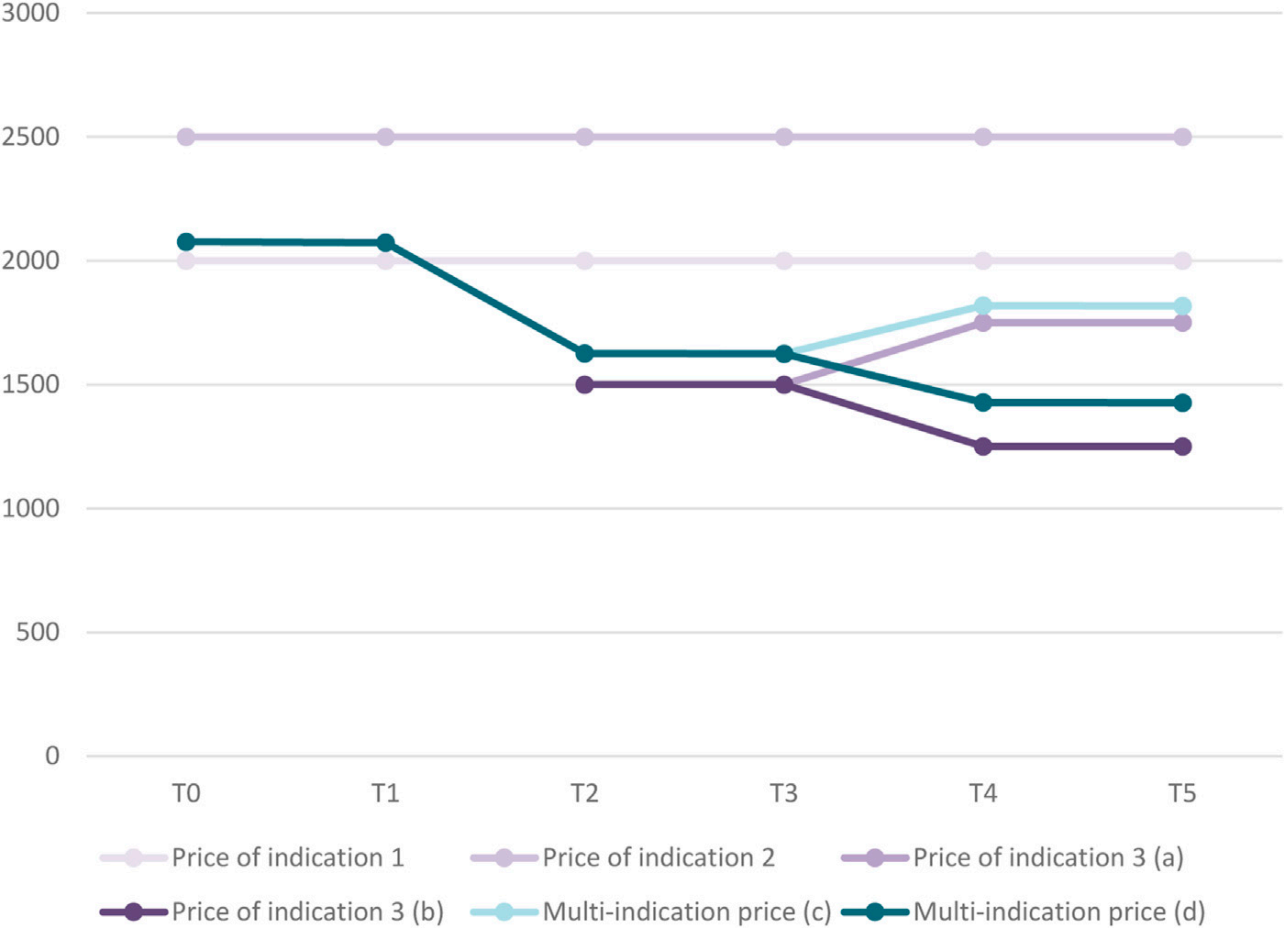
Evolution in price per indication and multi-indication price over time for case study.Legend: (a) Scenario in which price of indication 3 increases over time. (b) Scenario in which price of indication 3 decreases over time. (c) Based on scenario in which price of indication 3 increases over time. (d) Based on scenario in which price of indication 3 decreases over time.

Figure 3 displays the evolution in the price for each indication and in the multi-indication price over time for our hypothetical medicine. At time point TO, the volume- and value-weighted average price based on the two initial indications amounts to €2,077. The Figure also shows how this multi-indication price changes when a third indication is launched in T2 and when real-world evidence of the value of the medicine in the third indication becomes available in T4.

### 3.5 MIP procedural pathway

**FIGURE 4:**
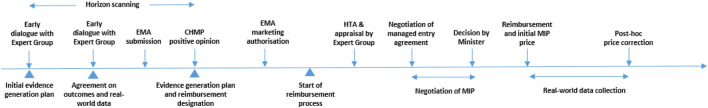
Procedural pathway for implementing MIP model 5 in Belgium. Notes: CHMP, Committee for Medicinal Products for Human Use; EMA, European Medicines Agency; HTA, health technology assessment; MIP, multi-indication pricing.

Figure 4 proposes a procedural pathway for implementing MIP model 5 in Belgium. This pathway starts with horizon scanning in order to gain insight into forthcoming medicines and their indications. Early dialogue with an expert group (consisting of representatives from the payer, pharmaceutical company, clinicians, data experts and patients) serves to reach agreement on an “evidence plan for the medicine”, which specifies which evidence is to be provided by the applicant, timelines and data sources; and how this evidence is to be evaluated. This dialogue also involves a discussion on real-world data and evidence generation to prepare the local infrastructure and data access in time. The order in which indications receive a positive opinion from the Committee for Medicinal Products for Human Use of the European Medicines Agency is also the order in which the payer considers indications for reimbursement (=“reimbursement designation”). The initial price of the medicine and its adjustments over time are then determined according to the specifications of the MIP model in the context of a managed entry agreement.

### 3.6 Prerequisite conditions for MIP implementation

For a MIP model to be successfully implemented in Belgium (or in other countries), a number of prerequisite conditions need to be satisfied. For instance, stakeholders need to move away from a static approach to pricing medicines and adopt a dynamic approach under which the price of a medicine decreases or increases during the lifecycle depending on its initial and real-life performance in multiple indications. As a result, the budget impact associated with the medicine is likely to change over time. Furthermore, a data infrastructure needs to be in place, which allows to collect real-world evidence on a medicine. This also requires stakeholders to agree on the funding and governance of such a data infrastructure. Moreover, it should be noted that the implementation of MIP for medicines may actually increase the administrative burden for the payer, company and physicians who contribute to collecting, analysing and appraising data on the medicine. This involves, for example, agreement about how the value of a medicine is measured and how the price of a medicine is adapted to its value.

## 4 Conclusion

When implementing a MIP model for medicines, policy and decision makers need to consider multiple facets which relate to the choice for uniform or differential pricing, price adjustment based on volume and/or value, proactive or retroactive dynamic pricing. Given that there tends to be uncertainty about the volume and value of a medicine for its indications, a sample of Belgian stakeholders chose a MIP model that accounts for the volume and value of the medicine in each indication at launch and during its lifecycle. Further research and real-life application of MIP models is needed to inform the further development of models calculating multi-indication medicine prices that incite value for money and competition, encourage monitoring and evaluation of medicine performance, promote rational use of medicines, and reduce complexity and administrative burden.

## Data Availability

The original contributions presented in the study are included in the article/Supplementary Material, further inquiries can be directed to the corresponding author.
